# Assessing the Impact of Anthropic Pressures on Aquatic Macroinvertebrates: A Functional Trait Approach in the Irtysh River Watershed

**DOI:** 10.3390/biology12101315

**Published:** 2023-10-08

**Authors:** Fei Liu, Fangze Zi, Xinyue Wang, Honghui Zeng, Bin Huo, Chengxin Wang, Jianmin Ge, Shengao Chen, Baoqiang Wang

**Affiliations:** 1 Tarim Research Center of Rare Fishes, College of Life Sciences and Technology, Tarim University, Alar 843300, China; liufei01@ihb.ac.cn (F.L.); 10757213076@stumail.taru.edu.cn (F.Z.); 120050007@taru.edu.cn (X.W.); 10757203067@stumail.taru.edu.cn (C.W.); 10757212140@stumail.taru.edu.cn (J.G.); 2 Institute of Hydrobiology, Chinese Academy of Sciences, Wuhan 430072, China; zhh@ihb.ac.cn; 3 College of Fisheries, Huazhong Agricultural University, Wuhan 430070, China; huobin@mail.hzau.edu.cn

**Keywords:** the Irtysh River Basin, functional traits, macroinvertebrate, urbanization

## Abstract

**Simple Summary:**

This study investigates the evolutionary dynamics of macroinvertebrate functional traits within the Irtysh River Basin under the influence of urbanization. Through comprehensive field assessments and data analysis, we document a significant transition process toward pollution-tolerant functional characteristics in macroinvertebrates, coinciding with the rapid pace of urban development. Simultaneously, the community undergoes a gradual transition, marked by an increase in pollution-tolerant taxa. Urbanization-induced environmental pollution and waste discharge emerge as prominent catalysts, accelerating the observed changes in macroinvertebrate communities and functional traits. Our findings underscore the critical role of anthropogenic factors in aquatic ecosystems and highlight the imperative of strategic management strategies to mitigate these effects. This study contributes to a deeper understanding of the intricate interplay among urbanization, environmental change, and benthic organism responses, providing essential insights for sustainable river management amidst urban expansion.

**Abstract:**

Little is known about how changes in the biodiversity and functional traits of macroinvertebrates in rivers respond to the responses of anthropic pressures and their driving factors. Macroinvertebrates were sampled at 17 sites in the Irtysh River Basin and classified macroinvertebrates into 10 traits and 38 categories between May and August 2022. Then, we performed R-mode linked to Q-mode (RLQ) analysis and calculated functional richness, evenness, divergence, and Rao’s quadratic entropy (RaoQ) for each site and community-weighted means for each trait category. Our results indicated that there were pronounced alterations in species variability in the urban region. Functional divergence indicated fierce competition among species and considerable niche overlap in the urban region. Functional evenness indicated that species abundance distribution and interspecific functional distance were not uniform in the urban region. Functional richness indicated that the urban region was the strongest region in terms of niche occupation, resource utilization, and buffering capacity for environmental fluctuations. Rao’s quadratic entropy showed that the trait difference of macroinvertebrates was the largest in all regions, which was caused by the gradient environmental difference. Research has revealed that urbanization significantly influences the evolutionary trajectory of macroinvertebrate fauna, culminating in an upsurge in pollution-tolerant species and a convergence of functional traits. We recommend strengthening the control of urban and industrial pollution and wise planning and management of land and water resources to mitigate the impact of anthropogenic destruction on habitat fragmentation in the Irtysh River Basin.

## 1. Introduction

Over the past 100 years, anthropic activity has significantly threatened riverine ecological integrity worldwide [1,2]. Stream ecosystems are confronted with increasing challenges due to the continuous expansion of anthropic activities, including the discharge of domestic sewage, elevated agricultural water consumption, industrial development, and the establishment of hydrological junctions [3,4,5,6]. Additionally, industrial, agricultural, and municipal wastewater have produced river eutrophication and organic compound pollution [7,8]. Therefore, the biological habitat gradually tends to a state of degradation [9]. Moreover, there has been an increase in the ecological stress index caused by the miniaturization and homogenization of the composition of biological communities [10,11,12]. Understanding the factors controlling the health of stream ecosystems is essential for conserving aquatic biodiversity and promoting a harmonious coexistence between humans and nature.

The Irtysh River Basin (IRB) originates from Zigertaidaban, north of Fuyun County, and flows to Kazakhstan after leaving the southern bay of Habahe County. It is the only cross-border river in China that flows into the Arctic Ocean. The IRB is a continental north-temperate cold basin with unique geographical and natural conditions. It maintains a relatively primitive aquatic ecological environment and is a suitable habitat for alpine cold aquatic organisms. The average annual temperature is only 4 °C, and the extreme temperatures in the basin can reach 40 °C and −51.5 °C. The annual sunshine hours of the IRB are approximately 2900 h, and the value has decreased in the past thirty years [13]. The airflow from the Atlantic Ocean entering the basin is uplifted by the mountains to form abundant precipitation. The rainfall is mainly concentrated in summer and autumn, accounting for 56% to 60% of the annual rainfall. Abundant water resources and precipitation improve the development of agricultural and animal husbandry resources along the river basin, which have grown into a primary source of local finance [14,15]. Moreover, the abundant water resources of the IRB can be widely used for hydroelectricity. Large water conservancy projects provide an essential electricity source for Xinjiang Province and broader regions, supporting industrial development and urbanization [16]. However, the construction of water conservancy facilities has reduced the flow and velocity in the IRB. Furthermore, overgrazing has reduced riparian vegetation cover and altered land utilization types, which has affected stream ecosystems by destabilizing the water circulation due to urbanization. Moreover, some cities, such as Beitun city and Altay city, near watercourses, which need large amounts of water resources in the context of accelerated urbanization needs, will accelerate the rate of watercourses drying up, the degradation of wetlands and the decline of aquatic biodiversity [17,18,19]. As one of the most critical Asian streams, the negative impact of anthropic activity on the aquatic ecosystem in the IRB remains to be comprehensively studied and evaluated.

As an important indicator used to measure the health and function of aquatic ecosystems, macroinvertebrates play a crucial role in nutrient cycling, organic matter decomposition, and sediment stability. The presence or absence of critical macroinvertebrates will have a combined effect on the entire ecosystem, affecting the abundance and distribution of other taxa [20,21]. Many researchers have reported establishing pollution tolerance values and studying the biodiversity of macroinvertebrates to scientifically evaluate stream system health [22,23]. They found that the extent of human interference in stream ecosystems is inversely proportional to the biodiversity of macroinvertebrate communities in freshwater ecosystems [24]. However, the index of abundance as a measure of biodiversity considers all groups equally, so this single index cannot identify subtle differences between hydrological units [25]. To avoid this problem, some researchers have proposed a method of functional diversity using biological traits such as habits, feeding behavior, and body size of macroinvertebrates to replace single indicators of biodiversity assessment [26,27]. Functional diversity refers to the value and range of species functional traits in a given community or ecosystem, and functional traits can reflect the difference in species functional diversity in biological communities [28,29]. Nevertheless, changes in environmental factors caused by anthropic disturbances are the leading cause of differences in the functional traits of macroinvertebrates. It was found that different levels of anthropic disturbance have different effects on macroinvertebrate community composition. For example, species of EPT taxonomic units decreased with increased anthropic activities, while pollution-tolerant species such as Chironominae increased [30,31]. In addition, the extent and type of macroinvertebrate communities affected by anthropic activities were related to geographic location and ecosystem characteristics, with macroinvertebrate communities in highly urbanized regions exhibiting lower diversity and abundance [32,33]. Therefore, we investigated macroinvertebrate community traits to further understand the complex interactions between macroinvertebrate taxa and their environment. Ultimately, it can lead to the designation of effective conservation strategies to mitigate the negative impacts of anthropic activity on aquatic ecosystems.

## 2. Materials and Methods

### 2.1. Field Sampling and Data Acquisition

**Figure 1:**
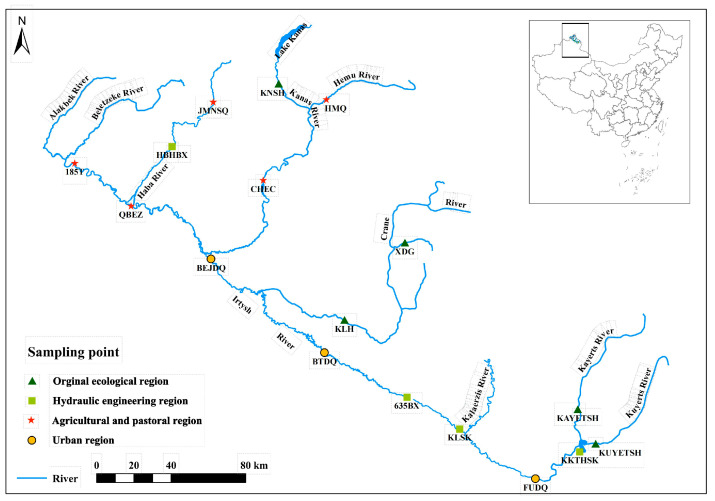
Map of the study sites showing the main land uses, the location of the sampling sites, and the IRB in China. The triangle indicates the original ecological region; the square indicates the region of hydraulic engineering; the asterisks indicate the agricultural and pastoral region; the circle indicates the urban region.

We studied the IRB located in Altay city, Xinjiang Uygur Autonomous Region of China. The main stream is 4248 km long, with a total length of 633 km in China and a basin area of 5.7 × 10^4^ km^2^ [19]. Considering the geographical and environmental characteristics, this study set up seventeen sampling sites in the IRB (Figure 1). Three sampling events were conducted at each site between May and August in 2022. On each sampling occasion, we measured the water temperature (WT, °C), atmospheric pressure (AP, Pa), dissolved oxygen (DO, mg/L), specific conductance (SPC, μs/cm), conductivity (C, μs/cm), salinity (SAL, ‰), pH, and oxidation-reduction potential (ORP, mV) in triplicate with a Multi-Parameter water quality Sonde (YSI 556MPS). According to the “Analytical Methods for Water and Wastewater Monitoring (4th edition)” standard, a 1 L plexiglass water collector was used to collect water quality samples. Additionally, we collected water samples for the analysis of total phosphorus (TP, mg/L), total nitrogen (TN, mg/L), and chlorophyll-a (Chl-a, μg/L) determinations [34].

We collected the river substrate using a D-shaped net (0.3 m mesh width, 500 μm mesh aperture) for 0.3 m^3^ at each sampling site. Samples were fixed in the field with 4% formaldehyde and sieved in the laboratory through a 60-mesh sieve. Samples were preserved in 70% ethanol, and macroinvertebrates were identified to at least the family or subfamily level [35].

By consulting published books and literature, we selected ten continuous biological traits to reflect the life history, resistance to the outside world, and physiological characteristics of macroinvertebrates [36,37,38]. These traits included trophic habit, habitat, stain resistance value, maximum size, reproduction, respiration technique, swimming ability, armoring, shape, and thermal preference. They were used to divide macroinvertebrates into 38 functional groups (Table 1). We utilized the fuzzy coding system to score the affinity of each trait unit. Each trait unit was assigned a score to describe the affinity of different forms in different variables. The score was proportional to the affinity, ranging from zero to three. For some families identified at a lower resolution level in taxonomy, we calculated the affinity score by adding the genus-level affinity scores that appeared in the region and readjusting the results from zero to three. We divided the subfamily or subfamily relationship of the family when we could not identify the family’s information in Diptera, Oligochaeta, and Hirudinae.

### 2.2. Data Analysis

We analyzed the data using a comprehensive approach comprising two methods to assess the macroinvertebrate functional traits. First, we performed R-mode linked to Q-mode (RLQ) analysis to assess the relationships between biological traits and environmental factors [36]. Second, we used community-weighted mean (CWM) analysis to identify the general distribution of functional biological shapes across each sampling site [37,38]. Finally, we used the functional richness (FRic), functional evenness (FEve), and functional divergence (FDiv) indexes and Rao’s quadratic entropy (RaoQ) analysis to evaluate the functional diversity [39,40].

#### 2.2.1. Physico-Chemical, Biological and Trait Analysis

We used the Pearson correlation method and the Benjamini–Hochberg (BH) adjustment to analyze the relationships between environmental factors and the family distribution of macroinvertebrates. First, we created two correlation coefficient matrices for species distribution and environmental factors at each point. Second, we adjusted the *p* value by the BH. Correlation was considered significant when the *p* value was less than 0.05. We decided to determine the association between the biological traits of macroinvertebrates by the association analysis (r > 0.85, *p* < 0.05). A data matrix was constructed using 38 functional group variables, and then we used association analysis.

#### 2.2.2. Functional Diversity and Community-Weighted Means

We utilized functional richness, functional evenness, and functional divergence to assess functional diversity. FEve represents the degree to which the functional traits of a single individual are evenly distributed over the ecological space. It is proportional to the degree of utilization of adequate resources within the ecological space. FDiv refers to the difference in the functional trait of individuals within the biological community, directly proportional to the degree of complementarity of the ecological niche and inversely proportional to the degree of resource competition. The distance variation between species was expressed by RaoQ analysis. The macroinvertebrate functional trait values were calculated from the community-weighted average trait index with the following equation:

(1) $$CWM=\sum_{i=1}^{n}P_{i}Trait_{i}$$

where $P_{i}$ is the abundance of taxon i in the community, and ${Trait}_{i}$ is the trait value of taxon i. This indicator holds that the functional trait of the most abundant species largely determines the functional trait of the community.

To assess the relationship between functional traits and environmental factors, we conducted RLQ analysis. For the subsequent statistical analysis of data, three data matrices were created: a matrix of water environmental variables (the R matrix), a macroinvertebrate taxa richness matrix (the L matrix), and a taxa functional trait matrix (the Q matrix). Pearson correlation analysis was used to assess the association among water environmental variables. When the Pearson associated coefficient reaches a certain threshold (r > 0.85, *p* < 0.05), the environmental factors associated with the coefficient must be eliminated. Before analysis, Hellinger’s transformation of the L matrix was required to reduce the influence of significant species and transform the data from the Q matrix to a log(x + 1) pattern. The R matrix was calculated by principal component analysis. The L matrix was analyzed by correspondence analysis, and the Q matrix was ranked using Hill–Smith. Finally, the covariates between environmental variables and species traits were maximized by RLQ ranking.

All analyses were performed in “R” version 3.5.1 (R Development Core Team, 2018). We used the following packages: ADE-4 for the RLQ analysis [41,42]; the PRIMER 7 package was used for the functional diversity index and CWM analysis [43]; and the “ideal” package was used for the indicator species analysis was implemented through the lands function.

## 3. Results

### 3.1. Environmental Factors and Family Distribution

**Figure 2:**
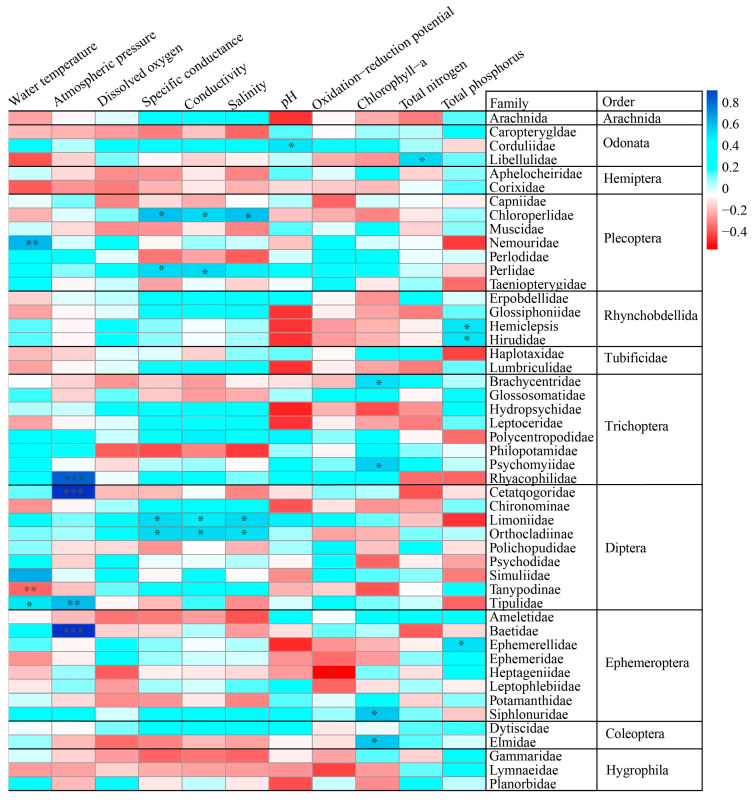
Heatmap showing the two sides of Pearson correlations between taxa abundance composition and environmental factors. *p* values were adjusted by Benjamini–Hochberg false discovery correction; * Benjamini–Hochberg adjusted 0.01 ≤ *p* < 0.05; ** Benjamini–Hochberg adjusted 0.001 ≤ *p <* 0.01; *** Benjamini–Hochberg adjusted *p* < 0.001.

A total of 2726 macroinvertebrate individuals belonging to 12 orders and 49 families were collected in this study, with Ephemeroptera accounting for 36.28% of the total sample size, followed by Diptera at 29.35%. The Ephemeroptera, Plecoptera, and Trichopetra (EPT) taxonomic units represented most species in the basin, accounting for 57.96%. Regarding taxonomic orders, Diptera was the richest, consisting of nine families, followed by Trichoptera, with eight families. The correlation analysis of taxon abundance and environmental factors (Figure 2) showed that Corduliidae of Odonata was significantly correlated with pH at 0.05. Chloroperlidae of Plecoptera, and Limoniidae and Orthocladiinae of Diptera were significantly positively correlated with SPC, C, and Sal at 0.05; Perlidae of Plecoptera was significantly positively correlated with SPC and C at 0.05. Hirudidae and Hemiclepsis of Rhynchobdellida and Ephemerellidae of Ephemeroptera were significantly positively correlated with TP at 0.05; Libellulidae of Odonata was significantly positively correlated with TN at 0.05. Simuliidae and Tipulidae of Diptera and Nemouridae of Plecoptera were significantly positively correlated with WT. Tipulidae and Cetatqogoridae of Diptera, Baetidae of Ephemeroptera, and Rhyacophilidae of Trichoptera all had highly significant positive correlations with AP at *p* values from 0.001 to 0.01. Psychomyiidae and Brachycentridae of Trichoptera, Siphlonuridae of Ephemeroptera, and Elmidae of Coleoptera were significantly positively correlated with Chl-a at 0.05. Heptageniidae of Ephemeroptera was significantly negatively correlated with ORP at 0.05.

### 3.2. Biological Traits

**Figure 3:**
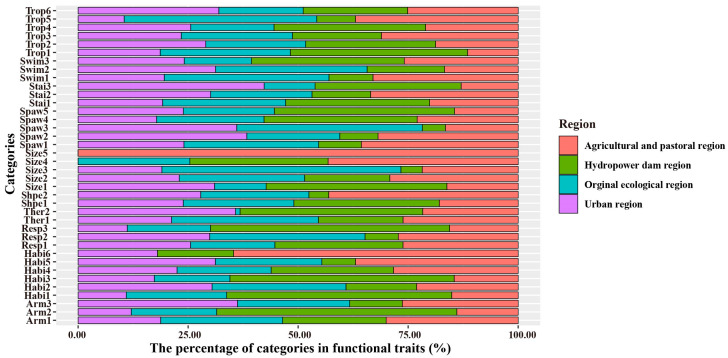
The percentage of categories in macroinvertebrate functional traits. See Table 1 for category abbreviations.

The proportion of functional traits of macroinvertebrate communities in different regions of the IRB was analyzed (Figure 3). By comparing the four areas and finding that the trophic habit in the original ecological region was mostly scrapers, the habitat tended to clinger; the maximum size was 20–40 mm; the spawning type was mainly spawning; they mostly used gills as respiration, and the swimming ability was also between weak and medium. The trophic habits of macroinvertebrates in the agricultural and pastoral regions were mostly shredders; the habitat tended to clinger, similar to the original ecological region; the stain-resistant taxa and armor strength accounted for the highest proportion; and most of the reproduction types in the agricultural and pastoral regions were fixed states of isolated eggs. Most of the macroinvertebrates in the hydropower dam region were pastoral food collectors, which were sprawlers, swimmers, and divers in habitat preference. The maximum size was mainly distributed in the two intervals of 10–20 mm and greater than 40 mm. The swimming ability was the weakest, and the body type was primarily nonstreamline. The functional traits of macroinvertebrates in the urban region were more prominent, reflected as parasites, predators, and collector-filterers in trophic habits. Regarding the habitat preferences expressed as burrowers and climbers, it is worth noting that the region had primarily sensitive groups; the maximum size was distributed chiefly within 5–10 mm.

**Figure 4:**
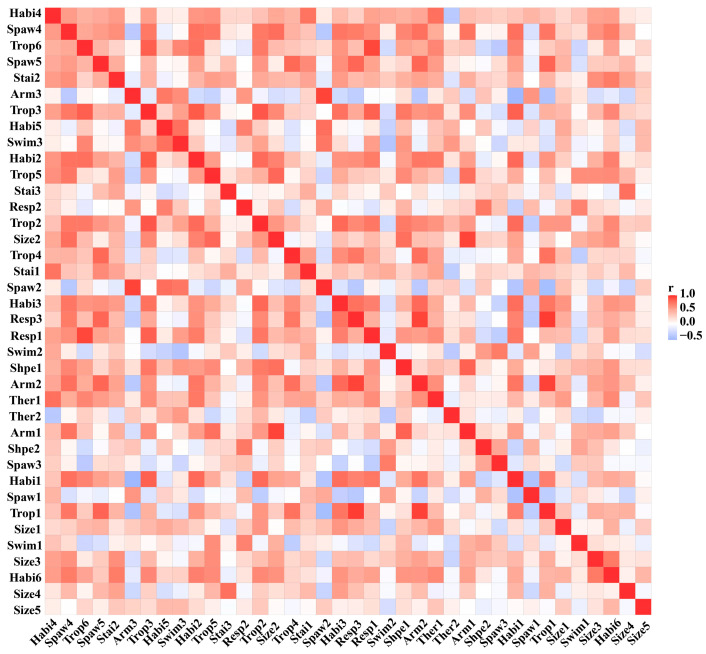
Correlation analysis between 38 categories of macroinvertebrate functional traits in the Irtysh River. See Table 1 for category abbreviations.

Figure 4 showed that parasites, collectors, and predators were positively associated with air respiration. Shredders were positively associated with tegument respiration, and the trophic habit gradually changed from air respiration to tegument respiration. Regarding feeding habits, the clingers and climbers were positively associated with the predators and shredders, and the scrapers were positively associated with the swimmers. There was a positive association between reproduction and swimming ability, and when swimming ability increased, reproduction tended to change from being free of isolated eggs to being cemented by isolated spawning. Notably, aquatic spawning had a solid positive association with all types of locomotion and attachment to substrates. Isolated eggs in the freestyle zone were negatively associated with parasites and air respiration in the trophic habit but were free of isolated eggs, and cemented isolated eggs were positively associated with gill respiration. Groups with maximum sizes in the five-to-ten-millimeter range were more suitable for narrow-temperature environments. When the maximum size was less than twenty millimeters, swimming ability was proportional to the maximum size. In comparison, when the maximum size was more than twenty millimeters, swimming ability was inversely proportional to the maximum size, and feeding habits were proportional to the maximum size. The pollution-tolerant organisms had a solid negative association with swimmers; pollution-sensitive organisms were positively associated with stenothermy; and with the increase in pollution resistance, the negative association with eurytherms also showed a state of gradual weakening.

### 3.3. Functional Diversity and Community Weighted Means

**Figure 5:**
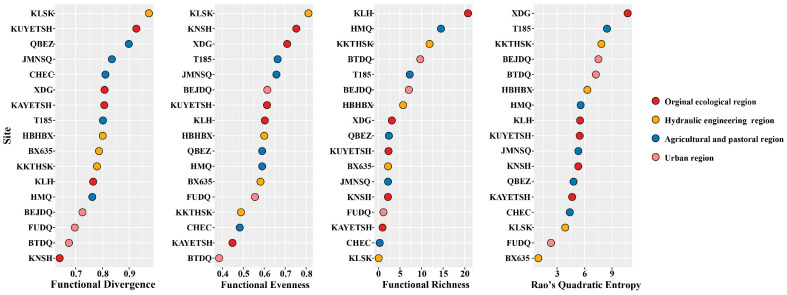
Functional diversity index and error distribution of each sample point in different regions. See Figure 1 for the classification of sampling sites.

For all the functional diversity indicators, the dot plot more intuitively shows the clustering of response variables (Figure 5). The results of FDiv showed that the top three sites with minimum sorts were FUDQ, BTDQ, and KNSH, while the sites with maximum sorts were KLSK, KUYETSH, and QBEZ. The FEve results indicated that the four sites with minimum sorts were BTDQ, KAYETSH, CHEC, and KKTHSK. In comparison, the maximum sites were KLSK, KNSH, and XDG, which decreased from agricultural and pastoral regions to hydroelectric dams and urban regions. The FRic results indicated that the top three sites with minimum values were KLSK, CHEC, and KAYETSH. In comparison, the maximum ranked values were at KLH, HMQ, and KKTHSKA. The value showed an increasing trend from agricultural and pastoral regions to hydroelectric dams and urban regions. The three sites with the maximum values were KLH, HMQ, and KKTHSKA, increasing from agricultural and pastoral regions to hydroelectric dams and urban regions. The three sites from RaoQ analysis rankings were BX635, FUDQ, and KLSK. The maximum rankings were found for XDG, T185, and KKTHSK, and the values fluctuated considerably between transition regions.

**Figure 6:**
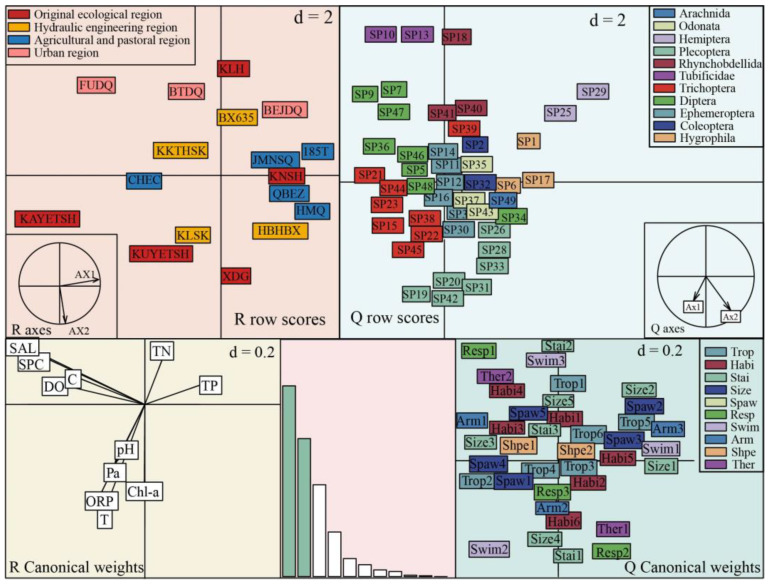
RLQ analysis results of functional traits of macroinvertebrates on the gradient of water environment variables. See Appendix A for codes of taxa-level Latin names. See Table 1 for category abbreviations.

We performed RLQ analysis sorting to explore the distribution pattern of macroinvertebrate functional traits in the IRB. The total slope inertia of the RLQ analysis was 1.679, and the first two axes extracted 64.6% of the initial total inertia. They indicated that the eigenvalues have a significant representation. The positive direction of axis 1 indicated changes in water environment variables such as TP, TN, DO, and SAL, which were primarily distributed among species of the taxonomic orders Libellulidae, Lymnaeidae, Aphelocheiridae, and Corixidae. The positively associated functional traits included shredders, isolated eggs and fixed state, medium stain resistance, and burrowers. Along axis I, the negative direction of the environment changed, mainly distributing taxonomic order species such as Polichopudidae, Simuliidae, Tanypodinae, Orthocladiinae, and Chironominae. Axis II was predominantly characterized by Chl-a, pH, and WT as the environmental gradient (Figure 6).

## 4. Discussion

This study assessed the functional traits of macroinvertebrates in four regions of the IRB. Our results indicated that anthropic activity accelerated the evolution of macroinvertebrate functional traits in the urban rural and hydraulic engineering regions. In contrast, macroinvertebrates in the original ecological region showed richer functional diversity, and the traits in this region were less disturbed.

Physicochemical variables within the aquatic environment hold promise as potential indicators for predicting community changes during assessments of the impacts of urbanization and habitat succession on macroinvertebrates [35,44]. We found that the Trichoptera was most abundant in the original ecological region and the hydraulic engineering region. Physicochemical variables showed the characteristics of low water temperature in original ecological region and high dissolved oxygen content in hydraulic engineering region. Based on the above, we predicted that low water temperature and high dissolved oxygen were the main factors that determine the density distribution of Trichopetra. This was consistent with a large number of typical studies [45,46]. Additionally, the density of EPT taxonomic units (Heptageniidae, Trichoptera, and Plecoptera) were reduced from the original ecological region to the urban region. Numerous studies have unequivocally demonstrated that urbanization processes pose significant threats to the ecological integrity of stream ecosystems within watersheds, resulting in the reduced presence of sensitive species, homogenization of food sources for higher trophic levels, and compromised stability of biological communities [47,48,49]. Simultaneously, the incremental expansion of the river channel in urban locales yielded diminished transparency and heightened sedimentation [50,51]. Jiang pointed out that using nitrogen fertilizers and pesticides is one of the critical factors contributing to river water quality pollution [50]. The elevation of TP and TN contents heightened stress resilience among macroinvertebrates, leading to the trend of increasing the population of fouling-tolerant species from the original ecological region to the urban region. This observation suggested an accelerated shift in the macroinvertebrate community owing to heightened human activities. Research has revealed that urbanization significantly influences the evolutionary trajectory of macroinvertebrate fauna, culminating in an upsurge in pollution-tolerant species and a convergence of functional traits [52,53].

The original ecological region exhibited distinctive functional traits among macroinvertebrate organisms, including scrapers, sprawlers, size of maturity at 5–10 mm, monoecious/asexual reproduction, pneumatic respiration, strong swimming ability, good armoring, streamlined shape, and eurythermal properties. Serving as a representative indicator of the river’s macroinvertebrate community health, scraper remained related to water quantity and water quality [54,55]. The prevalence of scraper-related functional traits in this region implied a correlation between the functional attributes of macroinvertebrate scrapers and the favorable environmental state, alongside the absence of pollution, in the water in the ecological region. This result coincides with the small proportion of Diptera and Coleoptera in this region. The sites within the original ecological region were situated at higher elevations, predominantly upstream of the IRB. Consequently, the cooler water temperatures prevailing in the upper river reaches, influenced by meltwater, aligned with the region’s prevalence of cold-adapted biological traits. Based on the above findings, we propose that there was an inevitable link between the speed of the species’ response to environmental pollution and water temperature. And when the water temperature increased, the response speed tended to decrease. At the same time, the content of eutrophication substances also confirm the conclusion.

In the agricultural and pastoral region, prevailing functional traits include shredders, strong swimming ability, size of maturity at 10–20 mm, aquatic spawning, gill respiration, good armoring, no streamlined shape, and stenothermy. The occurrence of pollution-tolerant taxa, notably Perlodidae and Orthocladiinae, indicated a moderate level of water pollution within the region. Furthermore, analyses of physico-chemical indicators within the agricultural and pastoral domains revealed elevated nitrogen and phosphorus levels attributable to the local livestock industry’s advancement. Specific locations, such as the QBEZ, lie near human habitation zones, where the release of domestic sewage compounds pollutes river segments [56,57]. However, the elevated abundance of avian species within agricultural and pastoral regions may stem from the heightened nutrient concentration in these waters, fostering algal and zooplankton proliferation and subsequently amplifying bird populations [58,59,60,61,62,63]. Smakhtin similarly identified an augmented prevalence of avian species within the macroinvertebrate fauna of urban rivers, typically correlated with water pollution and anthropogenic disruptions [64]. The mechanism through which functional traits react to environmental disturbances remains unclear and warrants further evaluation to refine the development of precise management strategies.

The functional traits within urban environments were predominantly characterized by shredders, sprawlers, medium stain resistance, size of maturity at 10–20 mm, aquatic spawning, gill respiration, strong swimming ability, good armoring, streamlined shape, and stenothermy. Multiple studies have demonstrated a close connection between numerous swimming macroinvertebrate organisms and urban industrial pollution. Given organisms’ heightened sensitivity to alterations in the aquatic environment, swimming macroinvertebrates can promptly evade polluted water sources [53,65,66]. Nonetheless, the current study challenges these conclusions, as strong swimming traits were prevalent across all four regions. Consequently, a hypothesis emerged that the elevated proportion of strong swimming in urban areas aligns with species adept at waterborne mobility. Moreover, a study by Utz revealed shifts in macroinvertebrate community structure attributed to water pollution and habitat degradation stemming from urbanization [67]. Nonetheless, instances arise where some macroinvertebrates displaying robust swimming traits emerge as the exclusive dominant species. This alignment coincides with the present study’s findings, demonstrating that robust swimming ability within macroinvertebrate organisms cannot singularly serve as an indicator for assessing water body pollution. Furthermore, macroinvertebrate organisms with robust swimming skills can harness energy to propel water currents through high-speed swimming, facilitating enhanced oxygen intake. This behavior is correlated with the diminished dissolved oxygen levels often observed in urban areas. Presently, both domestic and international researchers frequently employ pollution tolerance values as pivotal reference criteria for water environmental monitoring and assessment of water pollution status [68,69,70,71,72]. Regarding maturity, the biomass of pollution-tolerant moderate and high taxa within urban areas significantly surpassed that of the remaining three regions. Concurrently, an augmentation in pollution-tolerant taxa, exemplified by Tanyctolidae, Ophiuroidea, and additional pollution-indicator taxa, was observed at the FUDQ and BTDQ sites within urban areas. This overall trend implied heightened pollution levels within urban water. Urbanization has engendered a decline in diversity and tolerance among riverine macroinvertebrate fauna, primarily attributed to escalated nutrient concentrations and diminished substrate roughness [73].

The present study illustrated the functional traits of macroinvertebrates within the IRB with the acceleration of urbanization. The community has progressively transitioned into an ecological setting characterized by a prominent presence of pollution-tolerant taxa. Environmental pollution and waste discharge from urbanization processes have significantly influenced the acceleration of macroinvertebrate community transformation and the shaping of functional traits.

## 5. Conclusions

This study used RLQ analysis to assess the effect of anthropic activity on macroinvertebrate functional traits in the IRB. We found that the taxon composition of macroinvertebrates differed in regions disturbed by heavy anthropic activity compared with that in undisturbed regions. These species typically exhibited changes in functional traits associated with spawning and environmental adaptation. This was the first study of functional traits and environmental factors in macroinvertebrates within the Irtysh River basin in China. The results of our study can provide valuable information for the management and conservation of the IRB, and we can enhance watershed management by monitoring and assessing the impacts of anthropic activity on aquatic biodiversity. In addition, more effective methods for protecting and restoring damaged ecosystems can be explored. Examples include reducing pollutant discharge and human disturbance to restore aquatic habitats. Notably, we considered only the functional traits of macroinvertebrates without considering other biological taxa or ecosystem-level factors. Therefore, there may be some limitations in our study. To more deeply explore the impact of anthropic activity on ecosystems, future research can consider using multiple biological factors and multiple environmental factors to more fully assess ecosystem health.

## Figures and Tables

**Table 1:** Binary biological trait variables and categories of macroinvertebrate communities.

Trait	Category	Code	Abb
Trophic habit	Parasites	1	Trop1
	Predator	2	Trop2
	Collector-gatherer	3	Trop3
	Collector-filterer	4	Trop4
	Scraper	5	Trop5
	Shredder	6	Trop6
Habit	Burrower	1	Habi1
	Clinger	2	Habi2
	Climber	3	Habi3
	Sprawler	4	Habi4
	Swimmer	5	Habi5
	Divers	6	Habi6
Stain resistance value	Pollution-sensitive organisms < 3	1	Stai1
	Semi-tolerant to pollution 3–7	2	Stai2
	Pollution-tolerant organisms > 7	3	Stai3
Maximum size (mm)	5–10 mm	1	Size1
	10–20 mm	2	Size2
	20–40 mm	3	Size3
	40–80 mm	4	Size4
	>80 mm	5	Size5
Reproduction	Isolated eggs, free	1	Spaw1
	Isolated eggs, cemented	2	Spaw2
	Spawning	3	Spaw3
	Aquatic spawning	4	Spaw4
	Monogamy/Asexual reproduction	5	Spaw5
Respiration technique	Tegument	1	Resp1
	Gill	2	Resp2
	Air (spiracles, tracheae, plastrons)	3	Resp3
Swimming ability	Weak	1	Swim1
	Poor	2	Swim2
	strong	3	Swim3
Armoring	None	1	Arm1
	Poor	2	Arm2
	Good	3	Arm3
Shape	Streamlined	1	Shpe1
	Not streamlined	2	Shpe2
Thermal preference	Stenothermy	1	Ther1
	Eurytherm	2	Ther2

## Data Availability

The data supporting this study’s findings are available from the corresponding authors upon reasonable request.

## Appendix A

**Table A1:** Taxa-level Latin names and visualization of macroinvertebrate coding.

Number	Section
SP1	Lymnaeidae
SP2	Elmidae
SP3	Ephemerellidae
SP4	Siphlonuridae
SP5	Tipulidae
SP6	Gammaridae
SP7	Orthocladiinae
SP8	Leptoceridae
SP9	Chironominae
SP10	Lumbriculidae
SP11	Leptophlebiidae
SP12	Baetidae
SP13	Haplotaxidae
SP14	Ephemeridae
SP15	Psychomyiidae
SP16	Heptageniidae
SP17	Planorbidae
SP18	Hemiclepsis
SP19	Nemouridae
SP20	Taeniopterygidae
SP21	Philopotamidae
SP22	Brachycentridae
SP23	Polycentropodidae
SP24	Psychodidae
SP25	Aphelocheiridae
SP26	Muscidae
SP27	Potamanthidae
SP28	Capniidae
SP29	Corixidae
SP30	Ameletidae
SP31	Perlidae
SP32	Dytiscidae
SP33	Chloroperlidae
SP34	Cetatqogoridae
SP35	Libellulidae
SP36	Simuliidae
SP37	Caropterygldae
SP38	Glossosomatidae
SP39	Glossiphoniidae
SP40	Erpobdellidae
SP41	Hirudidae
SP42	Perlodidae
SP43	Corduliidae
SP44	Hydropsychidae
SP45	Rhyacophilidae
SP46	Polichopudidae
SP47	Tanypodinae
SP48	Limoniidae
SP49	Arachnida
